# Prevalence, Incidence and Determinants of Herpes Simplex Virus Type 2 Infection among HIV-Seronegative Women at High-Risk of HIV Infection: A Prospective Study in Beira, Mozambique

**DOI:** 10.1371/journal.pone.0089705

**Published:** 2014-02-24

**Authors:** Ivete Meque, Karine Dubé, Paul J. Feldblum, Archie C. A. Clements, Arlinda Zango, Fidelina Cumbe, Pai Lien Chen, Josefo J. Ferro, Janneke H. van de Wijgert

**Affiliations:** 1 Universidade Católica de Moçambique/Catholic University of Mozambique (UCM), Centro de Investigação de Doenças Infecciosas/Center for Infectious Disease Research (CIDI), Beira, Mozambique; 2 FHI 360, Clinical Sciences Unit, Durham, North Carolina, United States of America; 3 United States Military HIV Research Program (MHRP), Henry M. Jackson Foundation for the Advancement of Military Medicine, Inc. (HJF), Bethesda, Maryland, United States of America; 4 University of Queensland, Infectious Disease Epidemiology Unit, School of Population Health, Brisbane, Australia; 5 Amsterdam Institute for Global Health and Development (AIGHD) and Academic Medical Center of the University of Amsterdam, Amsterdam, The Netherlands; 6 University of Liverpool, Institute of Infection and Global Health, Liverpool, United Kingdom; Geisel School of Medicine at Dartmouth, United States of America

## Abstract

**Objectives:**

To estimate the prevalence, incidence and determinants of herpes simplex type 2 (HSV-2) infection, and associations between HSV-2 and incident HIV infection, among women at higher risk for HIV infection in Beira, Mozambique.

**Methods:**

Between 2009 and 2012, 411 women aged 18–35 years at higher risk of HIV acquisition (defined as having had two or more sexual partners in the month prior to study enrollment) were enrolled and followed monthly for one year. At each study visit, they were counseled, interviewed, and tested for HSV-2 and HIV antibodies.

**Results:**

The HSV-2 prevalence at baseline was 60.6% (95% CI: 55.7% –65.4%). Increasing age (aOR = 2.94, 95% CI: 1.74–4.97, *P*<0.001 and aOR = 3.39, 95% CI: 1.58–7.29, *P* = 0.002 for age groups of 21–24 and 25–35 years old respectively), lower educational level (aOR = 1.81, 95% CI: 1.09–3.02, *P* = 0.022), working full time (aOR = 8.56, 95% CI: 1.01–72.53, *P* = 0.049) and having practiced oral sex (aOR = 3.02, 95% CI: 1.16–7.89, *P* = 0.024) were strongly associated with prevalent HSV-2 infection. Thirty one participants seroconverted for HSV-2 (20.5%; 95% CI: 14.4% –27.9%) and 22 for HIV during the study period. The frequency of vaginal sex with a casual partner using a condom in the last 7 days was independently associated with incident HSV-2 infection (aOR = 1.91, 95% CI: 1.05–3.47, *P* = 0.034). Positive HSV-2 serology at baseline was not significantly associated with risk of subsequent HIV seroconversion.

**Conclusions:**

Young women engaging in risky sexual behaviors in Beira had high prevalence and incidence of HSV-2 infection. Improved primary HSV-2 control strategies are urgently needed in Beira.

## Introduction

Herpes simplex virus type-2 (HSV-2) infection is a chronic sexually transmitted infection (STI) and the main cause of genital ulcer disease (GUD) worldwide [1]–[3]. This infection constitutes a substantial public health problem in sub-Saharan Africa because it increases the risk of HIV acquisition two to four fold [2], [4]–[6]. HSV-2 and HIV have a synergistic relationship in that HSV-2 infection increases the susceptibility to and transmission of HIV, while HIV infection increases the susceptibility to HSV-2 infection and HSV-2 genital shedding [3], [7]–[9].

Reported risk factors for prevalent HSV-2 include older age [7], [8], [10], greater lifetime number of sexual partners [10], [11], working in bars and hotels [7], [12], prevalent HIV-1 infection, recent GUD or STI symptoms [10], [13], lower educational level [11], [14] and female gender [10], [13]. HSV-2 prevalence and incidence are generally high among young African women, particularly those who engage in risky sexual behaviors, including exchanging sex for money or goods [9], [12], [14], [15].

Studies in Southern Africa have shown variation in the prevalence of HSV-2 across diverse populations: 39.8%, 42.2% and 49.1% among male factory workers, urban and pregnant women in Zimbabwe, respectively [16]–[18]; 84% among female sex workers at truck stops in South Africa [15]; and 28% and 54.4% among GUD patients and male farm workers in Zambia, respectively [19], [20]. No population-based HSV-2 data are available for Mozambique.

Mozambique is a country severely affected by HIV/AIDS, with a 2009 national prevalence estimated at 11.5% among adults aged 15–49 years and 13.1% among women of the same age [21]. Sofala Province (of which Beira is the capital) had the highest HIV prevalence of 15.5% among adults aged 15–49 years and 17.8% among women [21]. Only 2% of women aged 15–49 years in Sofala province had two or more sexual partners in the last 12 months [21]. However, the HIV prevalence among this group was 32.6%, almost double the provincial rate among all women [22]. According to the National Strategic HIV and AIDS Response Plan 2010–2014, multiple sexual partners along with low condom usage are the main drivers of HIV acquisition in Mozambique and contribute to approximately 24–29% of all HIV new infections in the country [23].

The main aim of our study was to estimate HIV prevalence and incidence among HIV-seronegative women reporting two or more sexual partners in the past month in Beira, Mozambique, in preparation for clinical trials of new HIV prevention interventions. HSV-2 prevalence and incidence were also assessed given the synergies between the two infections. Here, we focus on the HSV-2 results and the association between HSV-2 infection and HIV seroconversion in this study population.

## Materials and Methods

### Ethics Statement

The study was approved by the National Ethics Committee (Comité Nacional de Bioética para a Saúde or CNBS) in Maputo, Mozambique, the Protection of Human Subjects Committee (PHSC) at FHI 360, Durham, NC, USA, and the Division of Human Subjects Protection of the Walter Reed Army Institute of Research (WRAIR), Washington, DC, USA. The current analysis was further approved by the School of Population Health Ethics Committee of the University of Queensland, Brisbane, Australia. Literate participants provided written informed consent for the cross-sectional survey and the prospective cohort study; illiterate participants provided a thumb print and brought a literate witness who signed the consent form on their behalf. Participants received approximately 5 USD reimbursement per scheduled study visit.

### Study Design and Population

Between December 2009 and September 2012, a total of 1,018 women aged 18–35 years reporting at least two sexual partners in the past month participated in a cross-sectional survey in Beira, Mozambique [22]. HIV-negative women were offered enrollment in a prospective cohort study and the first 411 eligible women who consented were enrolled. Additional eligibility criteria included Mozambican citizenship, unknown HIV status, no history of antiretroviral therapy or non-therapeutic injecting drug use, and not currently enrolled in another HIV-related research study. Detailed recruitment procedures have been described elsewhere [22]. Briefly, women were recruited from the community by outreach workers in places considered to be of potential high risk of HIV sexual acquisition, such as bars, barracks, kiosks, nightclubs, formal and informal markets, long-distance truck driver parking and secondary schools in and around the municipality of Beira. For virtually all women, enrollment into the longitudinal study occurred within 30 days of the cross-sectional survey visit. The small number of women who returned more than 30 days after their cross-sectional survey visit was required to repeat the survey procedures.

### Study Procedures

Participants were followed up monthly over a 12-month period. At baseline and at each monthly follow-up visit, trained nurses conducted a face-to-face interview to obtain information about demographics, socio-economic status, sexual and contraceptive behavior and presence of STI symptoms. Blood samples were collected at each study visit for HIV and HSV-2 testing. HIV testing was performed at each study visit while the participant was at the study clinic using a rapid HIV test algorithm whereas HSV-2 testing was done in batches on baseline samples and on study exit samples (final study visit or HIV seroconversion visit) if the baseline sample was negative. Blood samples from participants who seroconverted for HIV were also tested by CD4 cytometry and HIV-1 RNA PCR. A urine pregnancy test was performed at every study visit. All participants received pre- and post-HIV test and safer sex counseling as well as male and female condoms free of charge. Participants who reported STI symptoms were given syndromic treatment according to the Mozambican Ministry of Health guidelines and pregnant women were referred to antenatal care [24].

### Laboratory Methods

HSV-2 serological testing was performed at the UCM-CIDI Laboratory in Beira using the HerpeSelect 2 ELISA IgG assay (Focus Diagnostics, Cypress, CA, USA) following the manufacturer’s instructions. HIV rapid testing was performed in the research center in the presence of the study participant using the Determine HIV1/2 rapid test (Alere Medical Co. Ltd., Chiba, Japan) as a screening test and Uni-Gold (Trinity Biotech PLC, Bray, Ireland) as a confirmatory test. The SD Bioline HIV-1/2 3.0 rapid test (Standard Diagnostics Inc., Kyonggi-do, Korea) and/or ELISA were used to resolve discrepant rapid test results. Participants who tested HIV-positive received a CD4 count from the study clinic and were referred to public health centers to access HIV care. CD4 counts were determined on an EDTA blood sample at the clinical laboratory of the “Centro de Saúde Urbano Ponta-Gêa” using a BD FACSCalibur flow cytometry platform. Pregnancy testing was done by rapid urine hCG pregnancy test (Healthease Preg n Care, NEOMED IPA, Tzaneen, South Africa).

### Statistical Analysis

All data were recorded on standardized case report forms that were double-entered into a database. Data were analyzed using STATA software version 11.2 (Statacorp, College Station, TX, USA).

Categorical variables were expressed as percentages, and continuous variables as medians with inter-quartile ranges (IQR). Baseline HSV-2 prevalence was calculated as the number of participants who tested positive for HSV-2 at cohort enrollment divided by the number of enrolled participants. HSV-2 incidence was calculated as the number of women who seroconverted for HSV-2 during follow-up divided by the total number of HSV-2 seronegative women at cohort enrollment [26].

Bivariable logistic regression models were used to assess determinants of prevalent baseline HSV-2 infection one at a time in two subgroups: demographic/socioeconomic factors and sexual behavioral factors. All factors associated with HSV-2 infection at p≤0.20 in the bivariable analyses were included in explanatory multivariable logistic regression models. The first multivariable model included demographic/socioeconomic factors only (Model I) and the second model sexual behavior factors only (with age forced into the model due to its importance as a confounder: Model II). Finally, variables with p≤0.20 in Models I and II were combined into a final multivariable model (Model III). The same modeling approach was used to assess determinants of HSV-2 incidence (comparing women who seroconverted for HSV-2 to those who remained negative, and excluding those who tested HSV-2 positive at baseline) with HSV-2 seroconversion as the outcome.

A survival analysis using Kaplan-Meier curves and the log-rank test was used to compare the HIV seroconversion curves of women who were HSV-2 seropositive and seronegative at baseline. Bivariable analysis with Cox proportional hazard models was used to calculate hazard ratios (HR) and 95% confidence intervals (CI) for baseline HSV-2 status and time-varying HSV-2 status during follow-up as a determinant of HIV acquisition. The proportional hazard assumption was tested and was valid. The two models (one for baseline HSV-2 status and one for time-varying HSV-2 status) had similar goodness of fit based on martingale residuals.

## Results

### Characteristics of the Study Population

**Figure 1:**
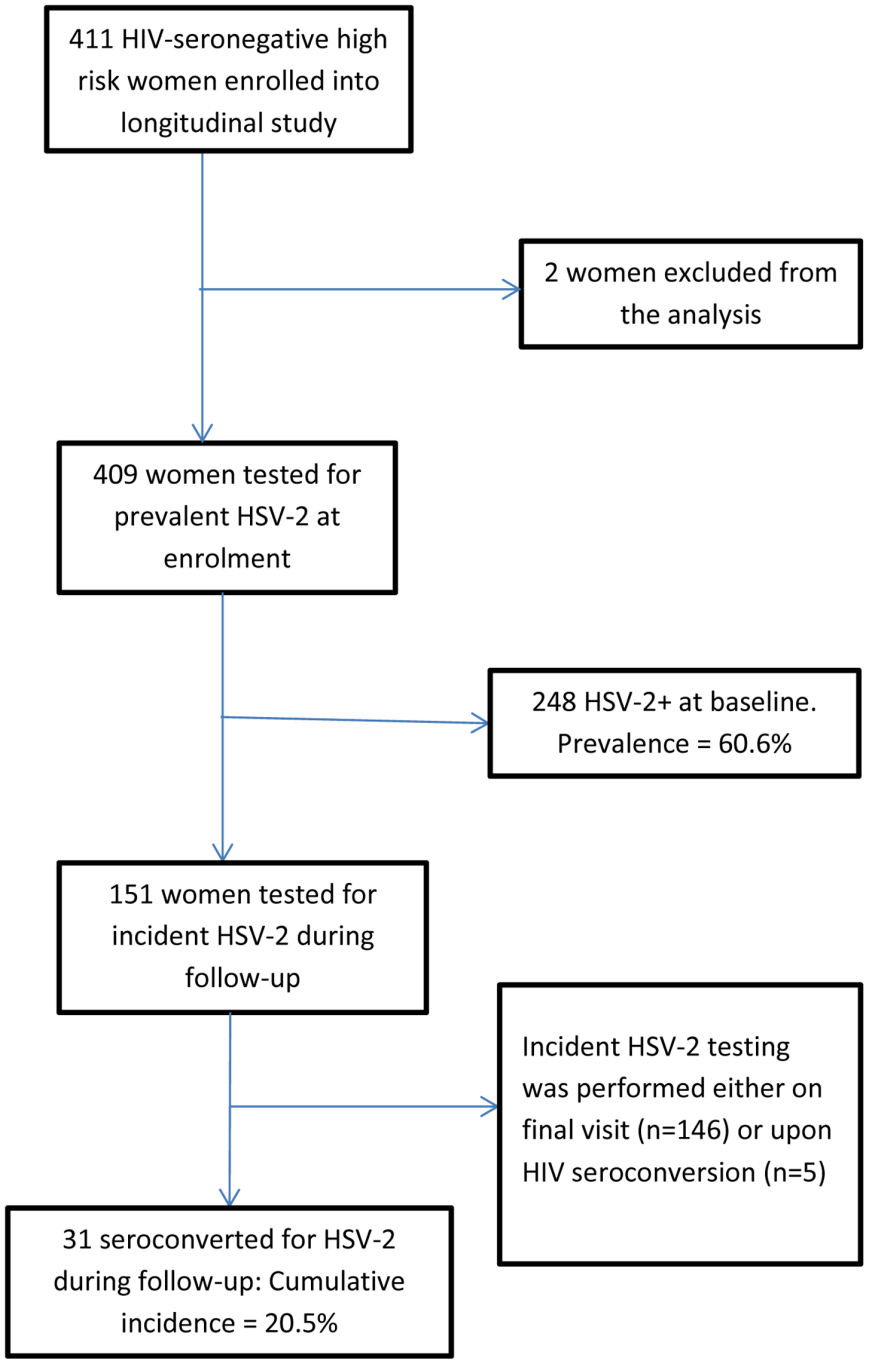
Participant flow diagram describing numbers enrolled, numbers tested for HSV-2 prevalence, and numbers included for HSV-2 incidence.

The first 411 HIV-negative cross-sectional survey participants who consented were enrolled in the prospective cohort study. Two women were excluded from all analyses: one woman had missing data at baseline and another did not meet the inclusion criterion of two or more sexual partners in the last month (Figure 1). The overall retention rate in the cohort study was 80%.

The median age of the participants was 21.0 years (IQR 19–24 years). About half of the participants (50.5%) were single and 56.0% had completed secondary school (grade 6–9). The majority of women (72.6%) were unemployed and 66.2% had no income in the last month. Forty three percent of women had been pregnant at least twice and 65.5% did not use a method of family planning. One third of the participants (33.3%) had sex for the first time at age 15 years or younger. More than two-thirds of women (70.4%) reported two sexual partners in the last month with the remaining 29.6% reporting three or more sexual partners. The majority of women (94.1%) reported to have a primary sexual partner, defined as a spouse, cohabitating partner, or a partner that the woman herself considered to be her most important partner. All other partners, including commercial partners, were defined as casual partners. About two-thirds of women (63.1%) reported that their primary partners had had sex with others in the last 6 months. Condom use was very low in this study population, with 79.9% and 57.0% of women reporting not having used condoms in the last month with a primary or casual partner, respectively. Oral and anal sex were relatively uncommon in this population (reported by 7.4% and 3.9% of women, respectively). The majority of the participants reported never having exchanged sex for money or goods (85.8%) or having been forced to have sex against their will (89.0%).

### Baseline HSV-2 Seroprevalence and Determinants

The HSV-2 seroprevalence at baseline was 60.6% (95% CI = 55.7–65.4%). In bivariable logistic regression analysis, HSV-2 seroprevalence increased significantly with age, age at first marriage, lower level of education, working full-time, having a higher income, having had two or more lifetime pregnancies and currently using hormonal contraceptives (Table 1). HSV-2 prevalence was also significantly higher among those whose primary partner was 30 years or older and those who did not use a condom during vaginal sex with a primary partner during the last month (Table 1). Prevalent HSV-2 infection was significantly lower in those who had sex for the first time at age 16–17 compared to those who were older.

**Table 1:** Determinants of baseline HSV-2 prevalence among 409 women, Beira, Mozambique.

Variable^a^	*n*	# HSV-2 positive/	Unadjusted OR	
		(Prevalence, %)	95% CI	*p*
Total	409^b^	248 (60.6)		
**Age group (years)**				
18–20	179	78 (43.6)	**Referent**	
21–24	145	102 (70.3)	3.07 (1.93–4.88)	<0.001
25–35	85	68 (80.0)	5.18 (2.82–9.51)	<0.001
**Age of first marriage (years)**				
Never married	205	106 (51.7)	**Referent**	
≤17	69	50 (72.5)	2.46 (1.36–4.46)	0.003
≥18	124	87 (70.2)	2.2 (1.37–3.52)	0.001
**Education**				
High school (grade 10–12)	132	67 (50.8)	**Referent**	
Secondary school (grade 6–9)	229	145 (63.3)	1.67 (1.08–2.59)	0.020
Primary school (grade1–5) or none	48	36 (75.0)	2.91 (1.39–6.08)	0.005
**Employment status**				
Unemployed	297	170 (57.2)	**Referent**	
Employed part time	92	59(64.1)	1.33 (0.82–2.17)	0.241
Employed full time	20	19 (95.0)	14.19 (1.88–107.43)	0.010
**Income last month (Mt)** c				
No income	268	152 (56.7)	**Referent**	
≥500	61	38 (62.3)	1.26 (0.71–2.23)	0.427
501–15,000	72	54 (75.0)	2.29 (1.27–4.11)	0.006
**Number of lifetime pregnancies**				
0	124	55 (44.4)	**Referent**	
1	108	58 (53.7)	1.46 (0.87–2.44)	0.156
≥2	177	135 (76.3)	4.03 (2.46–6.62)	<0.001
**Family planning use**				
None	268	152 (56.7)	**Referent**	
Hormonal contraceptives	95	68 (71.6)	1.92 (1.16–3.19)	0.012
Other d	46	28 (59.6)	1.19 (0.63–2.25)	0.599
**Age at first sexual intercourse (years)**				
≥18	102	68(66.7)	**Referent**	
16–17	163	85(52.2	0.54 (0.33–0.91)	0.021
≤15	136	91(66.9)	1.01 (0.59–1.74)	0.968
**Age of primary partner (years)**				
No PP	24	13 (54.2)	**Referent**	
Yes, aged 29 and younger	295	167 (56.6)	1.10 (0.48–2.55)	0.816
Yes, aged 30 and older	77	62 (80.5)	3.50 (1.31–9.33)	0.012
**Ever exchanged sex**				
No	350	206 (58.9)	**Referent**	
Yes	58	41 (70.7)	1.69 (0.92–3.08)	0.090
Forced to have sex in the last month				
No	363	224 (61.7)	Referent	
Yes	42	21 (50.0)	0.62 (0.33–1.18)	0.144
PP had sex with others in the last 6 months				
No	111	61 (55.0)	Referent	
Yes	258	168 (65.1)	1.53 (0.97–2.41)	0.066
Oral sex with PP in the last month				
No	378	224(59.3)	Referent	
Yes	30	23(76.7)	2.26 (0.95–5.39)	0.067
Frequency of vaginal sex with PP using condom in the last month				
Any use	82	41 (50.0)	Referent	
None	326	206 (63.2)	1.72 (1.05–2.80)	0.030
Pain during intercourse				
No	322	203 (63.0)	Referent	
Yes	88	46 (52.3)	0.64 (0.40–1.03)	0.068

OR, odds ratio; CI, Confidence intervals; PP, primary partner; CP, casual partner;

HSV-2, Herpes simplex virus type-2.

The total number of missing answers is 74.

? The following variables were also assessed for their association with prevalent HSV-2 but were not significant at the p<0.2 level in bivariable models:

Categorical variables: number of sexual partners, number of new sexual partners, oral sex with CP last month, anal sex with PP last month, anal sex with CP last month and frequency of vaginal sex with CP using condom last month.

Continuous variables: frequency of vaginal sex with PP last month, frequency of vaginal sex with CP last month, frequency of vaginal sex with PP using condom last 7 days and frequency of vaginal sex with CP using condom last 7 days.

b Totals for the categories are not always equal to 409 because of missing data.

c 1 USD = 29.70 Meticais (Mt) as per 29/03/2013 (Millennium bim Bank).

d Other Condoms/Copper IUD/Traditional.

In the final multivariable model, age was most strongly associated with baseline HSV-2 prevalence (adjusted odds ratio (aOR) = 2.94, 95% CI: 1.74–4.97, *P*<0.001 and aOR = 3.39, 95% CI: 1.58–7.29, *P* = 0.002 for age groups of 21–24 and 25–35 years old respectively). Other determinants of HSV-2 prevalence were having had secondary school (grade 6–9) compared to high school (grade 10–12) education (aOR = 1.81, 95% CI: 1.09–3.02, *P* = 0.022), working full-time (aOR = 8.56, 95% CI: 1.01–72.53, *P* = 0.049) and having practiced oral sex (aOR = 3.02, 95% CI: 1.16–7.89, P = 0.024) (Model III, Table 2). Having a primary partner who had sex with others in the last 6 months was not significant (aOR = 1.67, 95% CI: 0.99–2.80, *P* = 0.053). Conversely, having been forced to have sex in the last month was associated with reduced HSV-2 prevalence (aOR = 0.31, 95% CI: 0.15–0.66, *P* = 0.003).

**Table 2:** Multivariable models of factors associated with HSV-2 prevalence in high risk women in Beira, Mozambique.

Variable	MODEL I *n = *402		MODEL II *n* = 404		MODEL III *n* = 406	
	Adjusted OR	*P*	Adjusted OR	*p*	Adjusted OR	*p*
	95% CI		95% CI		95% CI	
**Age group (years)**						
18–20	**Referent**		**Referent**		**Referent**	
21–24	2.68 (1.60–4.47)	<0.001	3.22 (1.91–5.44)	<0.001	2.94 (1.74–4.97)	<0.001
25–35	2.89 (1.36–6.13)	0.006	4.95 (2.22–11.02)	<0.001	3.39 (1.58–7.29)	0.002
**Age first marriage (yrs)**						
Never married	**Referent**					
17 or less (≤17)	0.98 (0.46–2.07)	0.959				
18 and older (≥18)	0.82 (0.44–1.52)	0.527				
**Education**						
Grade 10–12	**Referent**				**Referent**	
Grade 6–9	1.61 (0.97–2.66)	0.063			1.81 (1.09–3.02)	0.022
Grade1–5 or none	1.93 (0.83–4.49)	0.124			2.24 (0.94–5.36)	0.069
**Employment status**						
Unemployed	**Referent**				**Referent**	
Employed part time	1.03 (0.55–1.93)	0.934			0.93 (0.53–1.61)	0.793
Employed full time	5.21 (0.63–43.27)	0.127			8.56 (1.01–72.53)	0.049
**Income last month (Mt)^1^**						
>500	**Referent**					
≤500	0.81 (0.36–1.86)	0.625				
No income	0.87 (0.43–1.79)	0.708				
**# lifetime pregnancies**						
0	**Referent**				**Referent**	
1	1.30 (0.73–2.33)	0.379			1.11 (0.62–1.97)	0.725
≥2	2.20 (1.09–4.45)	0.028			1.72 (0.91–3.26)	0.094
**Family planning use**						
None	**Referent**					
Hormonal contraceptives	1.18 (0.66–2.10)	0.579				
Other^a^	1.28 (0.64–2.58)	0.489				
**Age at first sex (years)**						
≥18			**Referent**			
16–17			0.77 (0.42–1.39)	0.383		
≤15			1.48 (0.79–2.76)	0.222		
Don’t know			0.47 (0.10–2.29)	0.349		
**Age of PP (years)**						
No PP			**Referent**			
Yes, <30			0.77 (0.27–2.18)	0.629		
Yes, ≥30			0.93 (0.27–3.22)	0.916		
**Ever exchanged sex**						
Never			**Referent**		**Referent**	
Yes			1.60 (0.81–3.16)	0.177	1.66 (0.83–3.32)	0.154
**Forced to have sex in the last month**						
No			**Referent**		**Referent**	
Yes			0.38 (0.18–0.81)	0.012	0.31 (0.15–0.66)	0.003
**PP sex with others in the last 6 months**						
No			**Referent**		**Referent**	
Yes			1.52 (0.92–2.51)	0.104	1.67 (0.99–2.80)	0.053
Not applicable, no PP			0.68 (0.28–1.64)	0.391	0.91 (0.40–2.08)	0.819
**Oral sex with PP**						
No			**Referent**		**Referent**	
Yes			3.03 (1.17–7.80)	0.022	3.02 (1.16–7.89)	0.024
**Frequency vaginal sex with PP using condom in the last month**						
Any use			**Referent**		**Referent**	
None			1.61 (0.92–2.81)	0.094	1.47 (0.83–2.60)	0.189
**Pain during intercourse in the past 3 months**						
No			**Referent**		**Referent**	
Yes			0.55 (0.32–0.95)	0.031	0.62 (0.36–1.06)	0.081
R^2^	0.1203		0.1374		0.1586	
AIC	507.182		501.9356		492.8155	
BIC	575.1217		569.9597		560.9235	

R^2^, Coefficient of Determination; AIC, Akaike Information Criterion; BIC, Bayesian Information Criterion;

Model I refers to the multivariable analysis of demographic/socioeconomic factors only;

Model II refers to the multivariable analysis of sexual behavior factors only, adjusted for age;

Model III refers to the multivariable analysis of both subgroups using cut off of P<0.20 in Models I and II.

a ‘Other’ Condoms/copper IUD/Traditional methods.

### HSV-2 Incidence and Determinants

Of 161 women who were HSV-2 negative at baseline, 151 women were tested for HSV-2 during the follow-up period. Thirty one of them (20.5%) seroconverted for HSV-2 during follow-up. In bivariable and multivariable analyses, HSV-2 acquisition during follow-up was significantly associated with frequency of vaginal sex with a casual partner using a condom in the last 7 days (OR = 1.86, 95% CI 1.03–3.38, P = 0.041; aOR = 1.91, 95% CI: 1.05–3.47, P = 0.034). For women who were 21–24 years compared to women who were 18–20 years, the aOR for HSV-2 incidence was 2.36 (95% CI: 0.98–5.71, P = 0.057). (Model III; Table 3).

**Table 3:** Multivariable models of factors associated with HSV-2 incidence in high risk women in Beira, Mozambique.

	MODEL I *n = *146		MODEL II *n = *151		MODEL III *n = *151	
Variable^a^	aOR (95% CI)	*p*	aOR (95% CI)	*p*	aOR (95% CI)	*P*
**Age group (years)**						
18–20	**Referent**		**Referent**		**Referent**	
21–24	1.92 (0.73–5.06)	0.185	2.27 (0.92–5.58)	0.074	2.36 (0.98–5.71)	0.057
25–35	0.82 (0.17–3.94)	0.802	1.39 (0.34–5.67)	0.644	1.37 (0.34–5.55)	0.657
**Age of first marriage (years)**						
Never married	**Referent**					
17 or less(≤17)	0.40 (0.07–2.18)	0.290				
18 and older (≥18)	1.53 (0.47–4.92)	0.477				
**# lifetime pregnancies**						
0	**Referent**					
1	0.70 (0.15–2.72)	0.606				
≥2	0.51 (0.17–1.70)	0.271				
**Frequency of vaginal sex with a** PP in the last month*			1.00 (0.95–1.06)	0.956		
**Frequency of vaginal sex with a** **CP using a condom in the** last 7 days*			1.82 (0.94–3.52)	0.075	1.91 (1.05–3.47)	0.034
**Anal sex with CP last month**						
No			**Referent**			
Yes			2.59 (0.26–24.35)	0.421		
**R^2^**	0.052		0.055		0.0503	
**AIC**	157.13		156.89		153.6	
**BIC**	178.01		174.99		165.67	

aOR, Adjusted Odds Ratio; *Continuous variables; R^2^, Coefficient of Determination; AIC, Akaike Information Criterion; BIC, Bayesian Information Criterion; PP, Primary Partner; CP, Casual Partner.

a The following variables were also assessed for their association with incident HSV-2 but were not significant at the p<0.2 level in bivariable models:

Categorical variables: education, employment status, family planning use, age at first sexual intercourse (years), number of sexual partners in the last month, number of new sexual partners in the last month, age of PP (years), ever exchanged sex, forced to have sex in the last month, PP had sex with others last 6 months, oral sex with PP in the last month, anal sex with PP in the last month, anal sex with CP in the last month, frequency of vaginal sex with PP using condom in the last month and frequency of vaginal sex with CP using condom in the last month.

Continuous variables: frequency vaginal sex with CP using condom in the last 7 days, frequency of vaginal sex with PP in the last month and frequency of vaginal sex with CP in the last month.

### Associations between HSV-2 Infection and HIV Seroconversion

**Figure 2:**
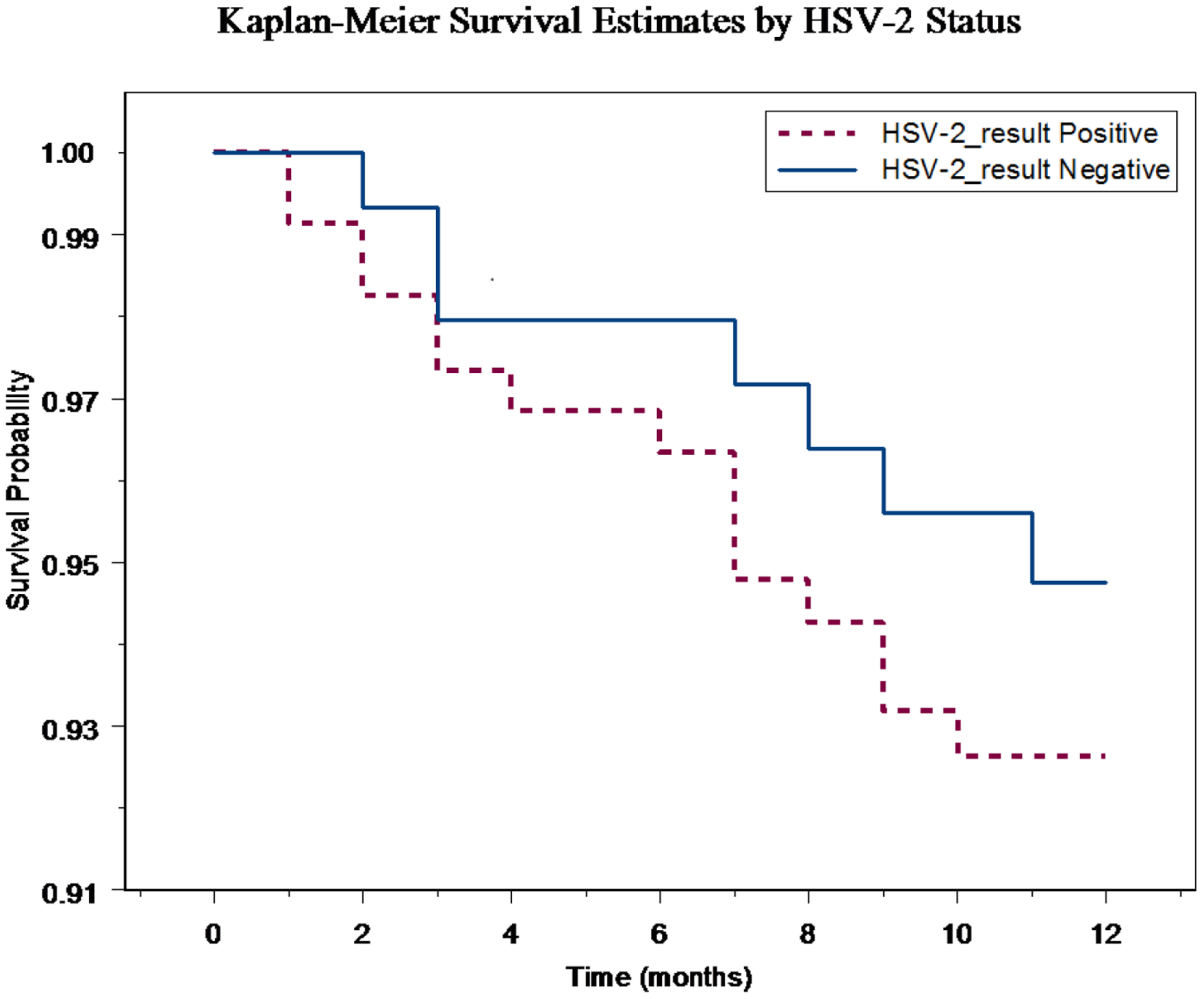
Kaplan-Meier curves for time to HIV seroconversion according to HSV-2 status at baseline.

Two hundred forty eight of 409 women were HSV-2 positive at baseline, of which 233 (94.0%) remained HIV-negative and 15 (6.1%) became infected with HIV during follow-up. Among the 22 women with HIV seroconversion during the 12-month follow-up period, 15 (68.2%) were HSV-2 positive at baseline, 2 (9.1%) seroconverted for HSV-2, and 5 (22.7%) remained uninfected during the follow-up period. Figure 2 shows Kaplan-Meier curves for time to HIV seroconversion according to HSV-2 status at baseline. The logrank test did not reach statistical significance (*P* = 0.406). A bivariable Cox regression model between HSV-2 status at baseline and subsequent HIV seroconversion showed a HR of 1.40 (95% CI: 0.57–3.44, P = 0.461). The relationship between HSV-2 serostatus at baseline and HIV seroconversion was not investigated further by multivariable analysis due to non-significant findings in bivariable analyses.

Of 31 women who seroconverted for HSV-2, 2 (6.5%) also seroconverted for HIV and 29 (93.6%) remained HIV negative during the follow-up period. A Cox regression model using time-varying HSV-2 as predictor was used to assess the associations between incident HSV-2 infection and incident HIV infection. The model showed a HR of 1.64 (95% CI: 0.61–4.46, *P* = 0.329).

## Discussion

This is the first study to assess prevalence, incidence, and determinants of HSV-2 infection in young, urban women in Mozambique at high risk of HIV infection. Women in this study had a high HSV-2 prevalence (ranging from 43.9% to 80.0% depending on the age group) and 20.5% seroconverted over a 12-month period, which is similar to rates found among high-risk women in other sub-Saharan African countries [7], [9], [27], [28]. These data suggest that interventions aimed at primary prevention of HSV-2, such as promotion of safer sexual behaviors, in young Mozambican women are urgently needed.

Increasing age was significantly associated with an increased HSV-2 seroprevalence and HSV-2 incidence, which is consistent with results of other African studies [7], [14], [27], [28]. Findings of the present study also add to the evidence that lower education, having a full time job, and having a primary partner who had sex with others in the last 6 months increased the risk of HSV-2 infection, although the latter not significantly. Most of the women in this study were working in informal settings, particularly in sales, food service and manual labor in bars, barracks and markets, and working full-time in these settings might be associated with exposure to multiple sexual partners. Studies among bar and hotel workers showed that these women are vulnerable because they engage in risky sexual behaviors [7], [9], [29].

Surprisingly, having been forced to have sex in the last month was associated with a lower risk of HSV-2 prevalent infection in this study population. The association between HSV-2 infection and being forced to have sex against one’s will could have been confounded by other factors such as age and other sexual behavioral factors; this finding should therefore be interpreted with caution.

Oral sex was associated with a 4-fold increased risk of baseline HSV-2 prevalence in this population. A study among high-risk (imprisoned) women in Iran found a significant association between anal and oral sex and a positive HSV-2 IgG test result, suggesting that HSV-2 can be transmitted through anal and oral sex [30]. Reporting oral sex might also be associated with engaging in exchanging sex for money or other high-risk sexual behaviors. Having practiced vaginal sex with a casual partner using a condom in the last 7 days was associated with an increase in risk of HSV-2 acquisition in our study population. This may suggest that there was inconsistent use of condoms among the participants and their casual partners or overreporting of condom use due to social desirability bias. Reporting of condom use might reflect sexual risk-taking behavior [12].

Various studies have suggested positive associations between prevalent and incident HSV-2 infection with incident HIV infection [5], [16], [31]. Our findings also show trends in that direction, but did not reach statistical significance. We believe that this is most likely due to limited statistical power (22 HIV seroconversions translates into 11% statistical power to detect an association between incident HSV-2 infection and incident HIV infection), but it is also possible that an association between HSV-2 and HIV infection does not exist in our study setting.

Other limitations of our study include the possibility of selection and social desirability biases, and a lack of generalizability to all women in Beira due to recruitment of high risk women with two or more sexual partners only. In addition, studies suggest that the HerpeSelect HSV-2 IgG assay has lower specificity in African than Western populations [25], [32], [33]. The authors of these studies have hypothesized that this may be due to cross-reactivity with HSV-1 or differences in circulating HSV-2 strains [25], [32], [33]. We did not confirm positive HerpeSelect HSV-2 results with another test to improve specificity, which may have resulted in some false-positive test results. The HSV-2 prevalence and incidence in our study may therefore have been overestimated.

In conclusion, this study confirms that HSV-2 prevalence and incidence among young women at risk for HIV in Beira, Mozambique, is high and is associated with increasing age and high risk sexual behaviors. In the absence of an HSV-2 vaccine, health campaigns among young people should promote condom use, reduction in numbers of sexual partners, and a delay of sexual debut. HSV-2 prevention and counseling should be given high priority on the Mozambican health agenda, and should be addressed along with HIV and other STIs as part of a comprehensive prevention package.

## References

[1] World Health Organization (2001) Herpes simplex virus type 2 programmatic and research priorities in developing countries. World Health Organization. London. Available: www.who.int/entity/hiv/pub/sti/pub9/en/. Accessed 2014 Jan 29.

[2] Celum C, Levine R, Weaver M, Wald A (2004) Genital herpes and human immunodeficiency virus: double trouble. Bulletin of the World Health Organization 82(6): 447–453. 15356938 PMC2622854

[3] Paz-Bailey G, Ramaswamy M, Hawkes SJ, Geretti AM (2007) Herpes simplex virus type 2: epidemiology and management options in developing countries. Sex Transm Infect 83(1): 16–22. 17098770 10.1136/sti.2006.020966 PMC2598582

[4] Freeman EE, Weiss HA, Glynn JR, Cross PL, Whitworth JA, et al (2006) Herpes simplex virus 2 infection increases HIV acquisition in men and women: systematic review and meta-analysis of longitudinal studies. AIDS 20(1): 73. 16327322 10.1097/01.aids.0000198081.09337.a7

[5] Serwadda D, Gray RH, Sewankambo NK, Wabwire-Mangen F, Chen MZ, et al (2003) human immunodeficiency virus acquisition associated with genital ulcer disease and herpes simplex virus type 2 infection: a nested case-control study in Rakai, Uganda. J Infect Dis 188(10): 1492–1497. 14624374 10.1086/379333

[6] Wald A, Link K (2002) Risk of human immunodeficiency virus infection in herpes simplex virus type 2-seropositive persons: a meta-analysis. J Infect Dis 185(1): 45–52. 11756980 10.1086/338231

[7] Kapiga SH, Sam NE, Shao JF, Masenga EJ, Renjifo B, et al (2003) Herpes simplex virus type 2 infection among bar and hotel workers in northern Tanzania: prevalence and risk factors. Sex Transm Dis 30(3): 187–92. 12616132 10.1097/00007435-200303000-00001

[8] Okuku HS, Sanders EJ, Nyiro J, Ngetsa C, Ohuma E, et al (2011) Factors associated with herpes simplex virus type 2 incidence in a cohort of human immunodeficiency virus type 1-seronegative Kenyan men and women reporting high-risk sexual behavior. Sex Transm Dis 38(9): 837–44. 21844740 10.1097/OLQ.0b013e31821a6225 PMC3157056

[9] Tassiopoulos KK, Seage G, Sam N, Kiwelu I, Shao J, et al (2007) Predictors of herpes simplex virus type 2 prevalence and incidence among bar and hotel workers in Moshi, Tanzania. J Infect Dis 195(4): 493–501. 17230408 10.1086/510537

[10] Mugo N, Dadabhai SS, Bunnell R, Williamson J, Bennett E, et al (2011) Prevalence of herpes simplex virus type 2 infection, human immunodeficiency virus/herpes simplex virus type 2 coinfection, and associated risk factors in a national, population-based survey in Kenya. Sex Transm Dis 38(11): 1059–66. 21992985 10.1097/OLQ.0b013e31822e60b6

[11] Arama V, Cercel AS, Vladareanu R, Mihai C, Mihailescu R, et al (2010) Type-specific herpes simplex virus-1 and herpes simplex virus-2 seroprevalence in Romania: comparison of prevalence and risk factors in women and men. Int J Infect Dis 14 (Suppl 3)e25–e31. 20106695 10.1016/j.ijid.2009.07.026

[12] Chohan V, Baeten JM, Benki S, Graham SM, Lavreys L, et al (2009) A prospective study of risk factors for herpes simplex virus type 2 acquisition among high-risk HIV-1 seronegative women in Kenya. Sex Transm Infect 85(7): 489. 19457873 10.1136/sti.2009.036103 PMC2813217

[13] Gottlieb SL, Douglas JM, Foster M, Schmid DS, Newman DR, et al (2004) Incidence of herpes simplex virus type 2 Infection in 5 sexually transmitted disease (STD) clinics and the effect of HIV/STD risk-reduction counseling. J Infect Dis 190(6): 1059–67. 15319854 10.1086/423323

[14] Watson-Jones D, Weiss HA, Rusizoka M, Baisley K, Mugeye K, et al (2007) Risk factors for herpes simplex virus type 2 and HIV among women at high risk in northwestern Tanzania: preparing for an HSV-2 intervention trial. J Acquir Immune Defic Syndr 46(5): 631–42. 18043318 10.1097/QAI.0b013e31815b2d9c PMC2643092

[15] Ramjee G, Williams B, Gouws E, Van Dyck E, De Deken B, et al (2005) The impact of incident and prevalent herpes simplex virus-2 infection on the incidence of HIV-1 infection among commercial sex workers in South Africa. Journal Acquir immune Defic Syndr 39(3): 333–9. 10.1097/01.qai.0000144445.44518.ea 15980695

[16] McFarland W, Gwanzura L, Bassett MT, Machekano R, Latif AS, et al (1999) Prevalence and incidence of herpes simplex virus type 2 infection among male Zimbabwean factory workers. J Infect Dis 180(5): 1459–65. 10515804 10.1086/315076

[17] Mbizvo EM, Msuya Sia E, Stray-Pedersen B, Chirenje MZ, Munjoma M, et al (2002) Association of herpes simplex virus type 2 with the human immunodeficiency virus among urban women in Zimbabwe. Int J STD AIDS 13(5): 343–8. 11972939 10.1258/0956462021925171

[18] Munjoma MW, Kurewa EN, Mapingure MP, Mashavave GV, Chirenje MZ, et al (2010) The prevalence, incidence and risk factors of herpes simplex virus type 2 infection among pregnant Zimbabwean women followed up nine months after childbirth. BMC Women’s Health 10(1): 2 doi: 10.1186/1472-6874-10-2 20064273 10.1186/1472-6874-10-2 PMC2819995

[19] Makasa M, Buve A Sandoy IF (2012) Etiologic pattern of genital ulcers in Lusaka, Zambia: has chancroid been eliminated? Sex Transm Dis 39(10): 787–91. 23001266 10.1097/OLQ.0b013e31826ae97d

[20] Heffron R, Chao A, Mwinga A, Sinyangwe S, Sinyama A, et al (2011) High prevalent and incident HIV-1 and herpes simplex virus 2 infection among male migrant and non-migrant sugar farm workers in Zambia. Sex Transm Infect 87(4): 283–8. 21459898 10.1136/sti.2010.045617

[21] Instituto Nacional de Saúde (INS), Instituto Nacional de Estatıstica (INE), and ICF Macro. (2010) National Survey on Prevalence, Behavioral Risks and Information about HIV and AIDS (INSIDA), Calverton, MD, USA. Maputo: INS, INE and ICF Macro. Available: http://www.measuredhs.com/pubs/pdf/AIS8/AIS8.pdf. Accessed 2014 Jan 29 January.

[22] Zango A, Dubé K, Kelbert S, Meque I, Cumbe F, et al (2013) Determinants of prevalent HIV infection and late HIV diagnosis among young women with two or more sexual partners in Beira, Mozambique. PLoS ONE 8(5): e63427. 23691046 10.1371/journal.pone.0063427 PMC3656941

[23] República de Moçambique, Ministério de Saúde, Conselho Nacional de Combate ao HIV/SIDA (2009) Plano Estratégico Nacional de Resposta ao HIV e SIDA 2010–2014. Available: http://www.cncs.org.mz/index.php/por/Publicacoes/Planos-e-Relatorios/Planos-do-CNCS. Accessed 2014 Jan 29.

[24] República de Moçambique, Ministério de Saúde, Programa Nacional de Controle as ITS/HIV/SIDA (2006) Guia para Tratamento e Controle das Infecções de Transmissão Sexual (ITS). Available: http://www.ensinoadistancia.edu.mz/sites/default/files/MANUAL%20CLINICO%20ITS.%202006_0.pdf. Accessed 2014 Jan 29.

[25] Hogrefe W, Su X, Song J, Ashley R, Kong L (2002) Detection of herpes simplex virus type 2-specific immunoglobulin G antibodies in African sera by using recombinant gG2, western blotting, and gG2 inhibition. J Clin Microbiol 40(10): 3635–40. 12354858 10.1128/JCM.40.10.3635-3640.2002 PMC130895

[26] Webb P, Bain C (2011) Essential Epidemiology: An Introduction for Students and Health Professionals. United States of America: Cambridge University Press, New York: 435.

[27] Weiss HA, Buvé A, Robinson NJ, Van Dyck E, Kahindo M, et al (2001) The epidemiology of HSV-2 infection and its association with HIV infection in four urban African populations. AIDS 15 (Suppl 4)S97–S108. 11686471 10.1097/00002030-200108004-00011

[28] Vandepitte J, Bukenya J, Weiss HA, Nakubulwa S, Francis SC, et al (2011) HIV and other sexually transmitted infections in a cohort of women involved in high-risk sexual behavior in Kampala, Uganda. Sex Transm Dis 38(4): 316–23. 23330152 PMC3920055

[29] Mgalla Z, Pool R (1997) Sexual relationships, condom use and risk perception among female bar workers in north-west Tanzania. AIDS Care 9(4): 407–16. 9337885 10.1080/713613167

[30] Asgari S, Chamani-Tabriz L, Asadi S, Fatemi F, Zeraati H, et al (2011) HSV-2 seroepidemiology and risk factors among Iranian women: a time to new thinking. Iran Red Crescent Med J 13(11): 818–23. 22737421 PMC3371894

[31] del Mar Pujades Rodriguez M, Obasi A, Mosha F, Todd J, Brown D, et al (2002) Herpes simplex virus type 2 infection increases HIV incidence: a prospective study in rural Tanzania. AIDS 16(3): 451–62. 11834958 10.1097/00002030-200202150-00018

[32] Van Dyck E, Buvé A, Weiss HA, Glynn JR, Brown DWG, et al (2004) Performance of commercially available enzyme immunoassays for detection of antibodies against herpes simplex virus type 2 in African populations. J Clin Microbiol 42(7): 2961–5. 15243045 10.1128/JCM.42.7.2961-2965.2004 PMC446279

[33] Delany-Moretlwe S, Jentsch U, Weiss H, Moyes J, Ashley-Morrow R, et al (2010) Comparison of focus HerpesSelect and Kalon HSV-2 gG2 ELISA serological assays to detect herpes simplex virus type 2 antibodies in a South African population. Sex Transm Infect 86(1): 46–50. 19837726 10.1136/sti.2009.036541 PMC2866038

