# Correction: Prevalence, Incidence and Determinants of Herpes Simplex Virus Type 2 Infection among HIV-Seronegative Women at High-Risk of HIV Infection: A Prospective Study in Beira, Mozambique

**DOI:** 10.1371/journal.pone.0100397

**Published:** 2014-06-13

**Authors:** The PLOS ONE Staff

The following information is missing from the Funding section: AZ was partially supported by a Fogarty International Center AIDS International Training and Research Program grant, National Institutes of Health, to the University of Pittsburgh (D43TW001038).
